# Fall-related hospitalization of patients in Iran

**DOI:** 10.1016/j.cjtee.2021.01.001

**Published:** 2021-01-14

**Authors:** Khalil Kimiafar, Maryam Farrokhi, Fereshte Manouchehri Monazah, Majid Khadem-Rezaiyan, Masoumeh Sarbaz

**Affiliations:** a Department of Medical Records and Health Information Technology, School of Paramedical Sciences, Mashhad University of Medical Sciences, Mashhad, Iran; b Department of Medical Informatics, Faculty of Medicine, Mashhad University of Medical Sciences, Mashhad, Iran; c Department of Community Medicine and Public Health, School of Medicine, Mashhad University of Medical Sciences, Mashhad, Iran

**Keywords:** Inpatient, Fall, Trauma

## Abstract

**Purpose:**

Trauma has been called the neglected disease of modern society. According to WHO, fall is the second major cause of trauma or deaths resulting from unintentional accidents. The aim of this study was to investigate the different types of fall according to International Statistical Classification of Diseases and Related Health Problems 10th Revision (ICD-10) in hospitalized patients visiting specialized accident and trauma hospitals of Mashhad, Iran.

**Methods:**

This was a cross sectional retrospective study performed between March 20, 2013 and March 20, 2014. The research population consisted of all medical records of patients for fall injuries in three specialized accident and trauma hospitals. ICD-10 was adopted to categorize all types of falls (w00-w19). The results obtained were analyzed by SPSS 16.

**Results:**

Altogether 7,448 cases were included. The codes w18 (fall on same level) and w09 (fall involving playground equipment) with the frequencies of 1,856 and 1,303, respectively in both genders had the maximum number of falls. The maximum percentage of mortality has been related to “fall on and from ladder”,” fall from cliff “and “fall on same level involving ice and snow”.

**Conclusion:**

As falls can cause irrecoverable injuries including mortality of people, thus health authorities and policymakers should take preventive measures given the causes of falls and the root of this type of injuries, so that the costs resulting from this cause and its injuries can be reduced.

## Introduction

Trauma has been called the neglected disease of modern society. This phrase may be more about describing trauma in developing countries, which invest little in public health programs or research on trauma.^1^^,^^2^ Trauma has negative effect on the victims, families, society, and social levels.^1^ According to WHO, fall is the second major cause of trauma or deaths resulting from unintentional injuries. Annually, around 424,000 deaths occur due to fall, 80% of which happen in medium and low income countries. A large number of deaths caused by falls occur in adults over 65 years old. According to global statistics, the rate of mortality caused by falls is high in “East Mediterranean Region”, which has been evaluated as 2.9 in every 100,000 falls.^2^ Falling is defined as an event, as a result of which a person falls on the ground or from other levels unintentionally. According to WHO fact sheet, revised in 2016, around 37 million falls have been so severe that they need medical attention each year. Fall has been the cause of over 17 million disabilities and the major cause of illness in adults over 65 years old, 15–29 years old and children ≤15 years old.^3^ Although fall injuries are usually reported in the elderly, various regional studies show that the young population (15–49 years old) and men are twice more likely to become affected by fall injuries, when compared with women.^2^^,^^4^^,^^5^ Around 40%–60% of falls lead to injuries, where 30%–50% of them are mild, 5%–6% are severe (except for fractures) and 5% belong to fractures. Ninety percent of hip fractures and wrist and 60% of head injuries have been developed due to falls. Falls are the most important common cause of death-associated injuries in over 75 years old.^6^

One of the important tools for investigation and comparison of the fall-related causes is created International Statistical Classification of Diseases and Related Health Problems 10th Revision (ICD-10). ICD-10 has been made to classify diagnoses and other health-related problems of patients hospitalized as alphabet-number codes. Using data coded according to the International Classification of Diseases statistically facilitates collecting morbidity data in a standard format and provides collection, storage and analysis of comparable data among countries.^7^ One hundred ten countries use the last edition of the international classification of diseases, i.e. ICD-10 for coding mortality and more than 20,000 scientific papers referred to it.^8^

In ICD-10, falls placed in categories (w00-w19), to indicate causes of falls including “on same level”, “from one level to another”, etc.^4^ Falls are more important in developing countries. For example, regional reports in Nepal and Iran showed that falls were the main cause of injuries especially in urban regions.^7^, ^8^, ^9^ According to a systematic study on epidemiology of injuries in Iran, falls (22.3%) ranked the second cause of damages.^10^ It has been estimated that around one-third of healthcare cost of accidents is related to falls, and two-thirds of its direct cost is related to hospitalization. It has been predicted that by 2050, the number and cost of injury caused by falls will grow by three times, and the cost of hospitalization will increase by 10 times.^11^ Falls can have other important complications such as functional, social, stress and concern for people,^12^ Regarding the effect of injury caused by accident on the number of years of life lost and the threat to the health of the country, and in most cases, the productive community, the study of the prevalence of various types of falls and their relationship with the demographic characteristics of individuals can help health planners and policymakers in planning and training the population to prevent incidence of such accidents. The aim of this study is to investigate the epidemiological features of different types of falls according to ICD-10 in hospitalized patients visiting specialized hospitals for accident across Mashhad City in 2014.

## Methods

This cross sectional retrospective study was performed between March 20, 2013 and March 20, 2014. The research population consisted of all medical records of patients for fall injuries in three specialized accident and trauma hospitals affiliated with Mashhad University of Medical Sciences (in the northeast of Iran).

Mashhad University of Medical Sciences is one of the biggest universities in Iran, with 7000 students in different fields of medicine and several academic hospitals. It is responsible for the health care of 3,001,184 inhabitants.

This study was only performed for patients hospitalized in the studied hospitals, and the outpatients were not included.

In the ICD-10, one chapter of the book (chapter 20) has been allocated to external causes of disease and mortality. In this study, according to ICD-10, all types of falls (w00-w19) were categorized. Then, based on these codes, the required information related to patients was extracted by trained experts. For each three character code, the fourth digit may be from 0 to 9, each of which specifies where the accident has happened. The fifth digit signified what the person has been doing during the accident. The results obtained from this research were analyzed by SPSS 16.

The study was approved by the research committee of the Mashhad University of Medical Sciences in Mashhad (Ethical code: IR. MUMS.REC.1394.750).

## Results

The total number of falls occurred in three specialized accident and trauma hospitals affiliated to Mashhad University of Medical Sciences was 7448, out of which 1.7% led to death. A total of 68% of the falls was related to men and the mean age of the injured was (35.1 ± 24.2) years. The codes w18 (fall on same level) and w09 (fall involving playground equipment) with the frequencies of 1856 and 1303, respectively in both genders had the maximum number of falls. The maximum percentage of mortality has been related to w11, w15 and w00 with 7.9%, 7.7% and 6.5%, respectively (Table 1). A significant difference existed between cause of falls and age (*p* < 0.001). Also, there was a significant difference between cause of falls and gender (*p* < 0.001). Falls occurred more frequent in men than in women. There was a significant difference between deaths due to fall and age (*p* < 0.001).

**Table 1:** Different types of fall in patients hospitalized in the studied hospitals, mean (SD) for “Age” and *n* (%) for other variables.

ICD-10 codes	Type of falls	*n* (%)	Gender		Age (years)	Mortality
			Men	Women		
W00	Fall on same level involving ice and snow	433 (5.8)	323 (74)	110 (25)	33.9 (18.5)	28 (6.5)
W01	Fall on same level from slipping, tripping and stumbling	345 (4.6)	296 (86)	49 (14)	38.4 (18.2)	0 (0)
W02	Fall involving ice-skates, skis, roller-skates or skateboards	161 (2.2)	125 (78)	36 (22)	31.0 (16.8)	1 (0.6)
W03	Other fall on same level due to collision with, or pushing by, another person	163 (2.2)	137 (84)	26 (16)	27.4 (19.4)	1 (0.6)
W04	Fall while being carried or supported by other persons	104 (1.4)	91 (88)	13 (12)	32.8 (19.6)	2 (1.9)
W05	Fall involving wheelchair	366 (4.9)	304 (83)	62 (17)	28.2 (11.8)	2 (0.5)
W06	Fall involving bed	98 (1.3)	53 (54)	45 (46)	34.8 (22.7)	1 (1.0)
W07	Fall involving chair	371 (5.0)	277 (75)	94 (25)	32.6 (20.7)	4 (1.1)
W08	Fall involving other furniture	346 (4.6)	198 (57)	148 (43)	36.7 (26.8)	4 (1.2)
W09	Fall involving playground equipment	1303 (17.5)	750 (57)	551 (43)	37.2 (26.2)	5 (0.4)
W10	Fall on and from stairs and steps	371 (5.0)	175 (47)	196 (53)	36.7 (28.6)	11 (3.0)
W11	Fall on and from ladder	38 (0.5)	25 (66)	13 (34)	47.6 (17.2)	3 (7.9)
W12	Fall on and from scaffolding	2 (0.0)	2 (100)	0 (0)	44.0 (11.3)	0 (0)
W13	Fall from, out of or through building or structure	96 (1.3)	75 (78)	21 (22)	22.9 (17.4)	5 (5.2)
W14	Fall from tree	30 (0.4)	28 (93)	2 (7)	34.4 (17.0)	0 (0)
W15	Fall from cliff	13 (0.2)	11 (85)	2 (15)	34.3 (19.4)	1 (7.7)
W16	Diving or jumping into water causing injury other than drowning or submersion	3 (0.0)	2 (67)	1 (33)	18.6 (9.7)	0 (0)
W17	Other fall from one level to another	1237 (16.6)	960 (78)	277 (22)	30.2 (21.6)	24 (1.9)
W18	Other fall on same level	1856 (24.9)	1105 (60)	745 (40)	39.4 (27.8)	29 (1.6)
W19	Unspecified fall	112 (1.5)	93 (83)	19 (17)	34.1 (19.7)	2 (1.8)
Total		7448 (100)	5030 (68)	2410 (32)	35.1 (24.2)	123 (1.7)

Table 2 shows the fourth and fifth digits of ICD-10 codes in Chapter 20 for codes (w00-w19), with the fourth digit representing the place of accident. In 88.2% of cases, place of accident was recorded as unknown. Fall in house (498 cases) and street (166 cases) had the largest frequency regarding place of accident. The fifth digit also represents the type of activity the person was doing during the accident. Similarly, in 80% of cases, activity of the person was recorded as unknown. As for those reported, sports activities (816 cases) had a large number.

**Table 2:** The place of occurrence and type of activity during the falls in patients hospitalized in the studied hospitals.

Fourth-digit of codes (W00–W19)	Place of occurrence	*n* (%)
0	Home	498 (6.7)
1	Residential institution	11 (0.1)
2	School, other institution and public administrative area	98 (1.3)
3	Sport and athletics area	41 (0.6)
4	Street and highway	166 (2.2)
6	Industrial and construction area	24 (0.3)
8	Other specified places^a^	42 (0.6)
9	Unspecified place	6568 (88.2)
Total		7448
Fifth-digit of codes (W00–W19)	Activity during fall	*n* (%)
0	While engaged in sports activity	816 (11.0)
1	While engaged in leisure activity	115 (1.5)
2	While working for income	495 (6.6)
3	While engaged in other types of work	8 (0.1)
4	While resting, sleeping, eating or engaging in other vital activities	42 (0.6)
8	While engaged in other specified activities	7 (0.1)
9	During unspecified activity	5965 (80.1)
Total		7448

a Other specified places include; Canal, Hill, Military Training, Mountain, Park (amusement) (public), Parking-lot and Parking-Place, Prairie, Railway Line, Water Reservoir and Zoo.

**Fig. 1:**
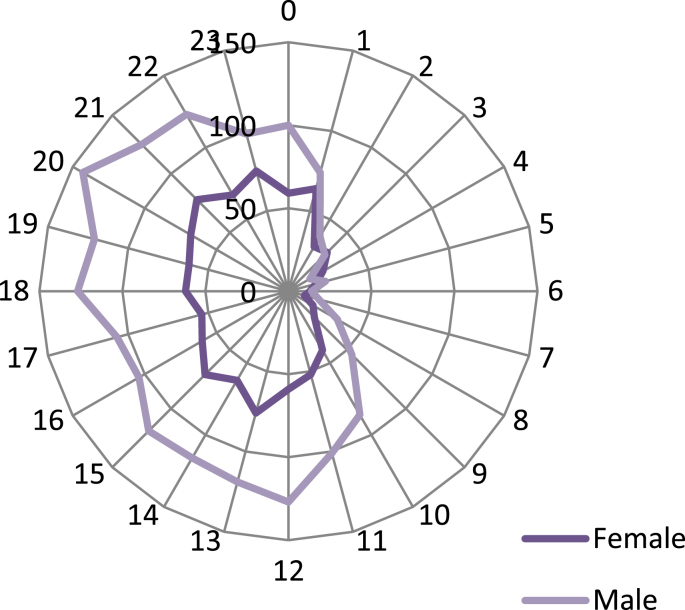
Frequency of number of falls according to hours of hospitalization across different hours of day.

**Fig. 2:**
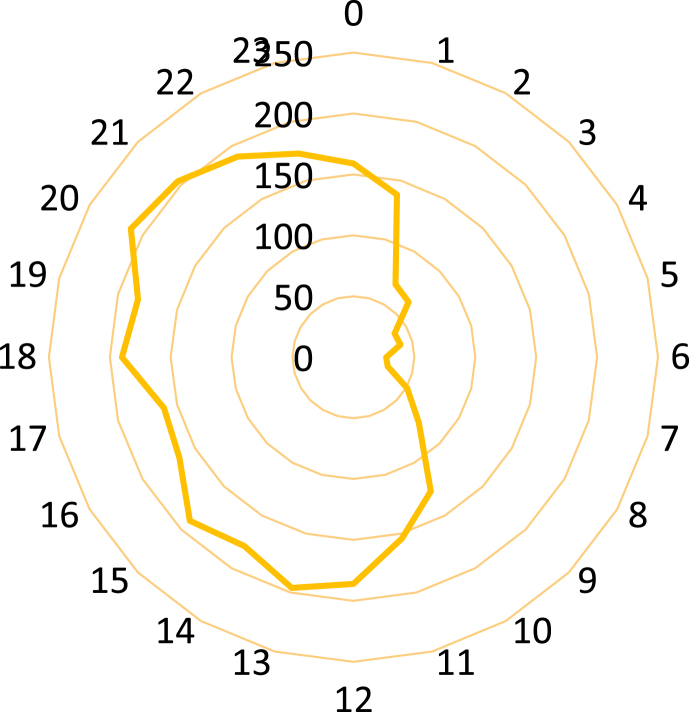
Frequency of number of falls based on hours of hospitalization across different hours of day by each gender.

The maximum and minimum number of falls based on hospitalization time of a day occurred at 8.00 p.m. and 6 a.m., respectively (Fig. 1). The number of hospitalization cases due to falls among men was larger than that of women across all hours of a day. The frequency of falls according to hospitalization hours of the person by each gender followed an almost identical trend (*p* = 0.15) (Fig. 2).

According to Table 3, which represents the number of falls occurring across different seasons of the year, the maximum number of cases occurred in summer and then spring with 2257 and 2175 cases, respectively, which in April the number was 796 cases. Different causes of falls showed a significant relationship with the month and season (*p* < 0.001).

**Table 3:** Types of fall by seasons in patients hospitalized in the studied hospitals.

ICD-10 Codes	Season, *n* (%)				Total
	Spring	Summer	Autumn	Winter	
**W00**	131 (30.5)	110 (25.4)	117 (27.0)	75 (17.3)	433
**W01**	76 (22.0)	126 (36.5)	94 (27.2)	49 (14.2)	345
**W02**	57 (35.4)	61 (37.9)	23 (14.3)	20 (12.4)	161
**W03**	50 (30.7)	64 (39.3)	33 (20.2)	16 (9.8)	163
**W04**	43 (41.3)	38 (36.5)	14 (13.5)	9 (8.7)	104
**W05**	125 (34.2)	129 (35.2)	68 (18.6)	44 (12.0)	366
**W06**	19 (19.4)	36 (36.7)	24 (24.5)	19 (19.4)	98
**W07**	108 (29.1)	122 (32.9)	86 (23.2)	55 (14.8)	371
**W08**	54 (15.6)	110 (31.8)	103 (29.8)	79 (22.8)	346
**W09**	497 (38.1)	338 (25.9)	250 (19.2)	218 (16.7)	1303
**W10**	96 (25.9)	114 (30.7)	90 (24.3)	71 (19.1)	371
**W11**	8 (21.1)	9 (23.7)	9 (23.7)	12 (31.6)	38
**W12**	0 (0)	1 (50.0)	0 (0)	1 (50.0)	2
**W13**	38 (39.6)	31 (32.3)	19 (19.8)	8 (8.3)	96
**W14**	11 (36.7)	14 (46.7)	5 (16.7)	0 (0)	30
**W15**	8 (61.5)	3 (23.1)	2 (15.4)	0 (0)	13
**W16**	1 (33.3)	1 (33.3)	0 (0)	1 (33.3)	3
**W17**	307 (24.8)	450 (36.4)	244 (19.7)	236 (19.1)	1237
**W18**	530 (28.6)	472 (25.4)	401 (21.6)	453 (24.4)	1856
**W19**	16 (14.3)	28 (25.0)	31 (27.7)	37 (33.0)	112
**Sum W00–W19**	2175 (29.9)	2257 (30.3)	1613 (21.7)	1403 (18.8)	7448

## Discussion

In this study, the total number of cases injured due to falls within a year across three hospitals for accident was 7448 cases. Based on the results of this study, the majority of injured people were men. In different studies dealing with investigation of the injuries caused by falls, 60%–70% of the injured were men,^9^^,^^13^ which is consistent with the results of our study. One of the possible reasons could be the social role or physiological conditions of men and high-risk behaviors including smoking, alcohol and drug abuse, which occur more often in men than in women.^14^ In this study, the major injured age group was 20–29 years (17.6%). About 50% of the fall cases were between 10 and 49 years old. Although injuries of falls are usually reported in the elderly, various regional studies have shown that the young population (15–49 years old) and men are twice more likely to be affected by the fall related injuries in comparison with women.^2^^,^^4^^,^^5^^,^^15^ In this study, among different types of falls, “fall on same level” had the maximum number of incidence. In a study conducted in five consecutive years in an Australian hospital, fall on same level has been introduced as the second most important external cause of injuries or falls.^16^ According to this study, the major age group of the injured caused by this cause (fall on same level) was related to children and the elderly. One possible reason could be disorders related to balance, walking, vision, hypotension and unsuitability of the house. Various studies have mentioned these factors as risk factors of falling in the elderly and children.^17^, ^18^, ^19^, ^20^, ^21^ In this study, “fall from stairs” was higher for women than for men. Possibly, things like the type of clothing and inappropriate shoes may have been effective. In other studies, “fall on same level” and “fall from stairs” were among the cases occurring mostly in women.^22^^,^^23^ Other codes showing considerable cases was w09 (fall involving playground equipment) with 1303 cases, standing second in highly frequent cases. In this study, 34% of the cases of this code was related to teenagers and children younger than 20 years old. According to Finlayson et al., in 2017, code w09 was the first rank of external causes of injuries among Canadian children.^16^ In another study, falling has also been introduced as one of the most important reasons of children hospitalization (0–14 years old).^24^ Maybe one of the reasons for fall from playground equipment is lack of safety of these instruments in parks, schools, and playgrounds. The study by Fragar indicated that falling from playing tools in children (0–14 years old) was 16,828 cases, and one of the most important reasons for this was the lack of safety features.^25^ According to WHO, falling is the second major cause of trauma or deaths resulting from unintentional injuries.^3^ In this study reported, 123 deaths was recorded due to falling, that about 50% of cases being related to deaths of the elderly. Different studies proposed that the underlying cause of death among the elderly was due to osteoporosis, and then fracture of the hip and eventually death.^26^, ^27^, ^28^ In this study, falling on the ground and following on ice and snow has dedicated the maximum percentage of death. This may be due to the fact that Mashhad City is an important and touristic city in Iran. In the first week of beginning of the year and during Nowruz holidays of March 2014, this city experienced snow and blizzard. As can be seen in Table 3, the number of falls in snow in April of this year was larger. Perhaps one of the main reasons of this event can be lack of preparation for incidence of this accident during this time. A research in England has stated that there is a significant relationship between falling on snow and ice and weekly air temperature. During times when snow and ice occur unexpectedly, hospitalization due to falling on ice and snow significantly increases due to lack of preparation of the people and the authorities of the city.^29^

In spite of the fact that coding of the external causes of injury in the studied hospitals is performed by skilled experts and the experts of health information management, not investigating the quality of coding in this research can be considered a limitation of this study. Another point that can be considered as a limitation in this research is the number of unknown cases in the part of the place and type of activity during accident. This shows that documentation of the medical records of these patients and taking history from them have not been performed completely.^30^

In conclusion, as falling can cause irrecoverable injuries including mortality of people, thus health authorities and policymakers should take preventive measures given the causes of falls and the root of this type of injuries, so that the cost resulting from these causes and its injuries can be shortened.

## Funding

Nil.

## Ethical statement

The study was approved by the research committee of the Mashhad University of Medical Sciences in Mashhad (Ethical code: IR. MUMS.REC.1394.750).

## Acknowledgments

The authors would like to thank the Research Deputy of 10.13039/501100004748Mashhad University of Medical Sciences, Mashhad, Iran for its support in this study (Code: 931787). We also appreciate the help of Clinical Research Development Unit of Akbar Hospital affiliated to Mashhad University of Medical Sciences.

## Declaration of competing interest

The authors state that there are no competing interest.
